# HLA-C Peptide Repertoires as Predictors of Clinical Response during Early SARS-CoV-2 Infection

**DOI:** 10.3390/life14091181

**Published:** 2024-09-19

**Authors:** Michael D. Olp, Vincent A. Laufer, Andrew L. Valesano, Andrea Zimmerman, Kenneth J. Woodside, Yee Lu, Adam S. Lauring, Matthew F. Cusick

**Affiliations:** 1 Department of Pathology, University of Michigan, 2800 Plymouth Rd Building 35, Ann Arbor, MI 48109, USA; 2 Sharing Hope of South Carolina, Charleston, SC 29414, USA; 3 Gift of Life Michigan, Ann Arbor, MI 48108, USA; 4 Academia Invisus LLC, Ann Arbor, MI 48107, USA; 5 Division of Nephrology, Department of Internal Medicine, University of Michigan, Ann Arbor, MI 48109, USA; 6 Division of Infectious Diseases, Department of Internal Medicine and Department of Microbiology and Immunology, University of Michigan, Ann Arbor, MI 48109, USA

**Keywords:** HLA, SARS-CoV-2, antigenicity, bioinformatics, computational biology

## Abstract

The human leukocyte antigen (HLA) system plays a pivotal role in the immune response to viral infections, mediating the presentation of viral peptides to T cells and influencing both the strength and specificity of the host immune response. Variations in HLA genotypes across individuals lead to differences in susceptibility to viral infection and severity of illness. This study uses observations from the early phase of the COVID-19 pandemic to explore how specific HLA class I molecules affect clinical responses to SARS-CoV-2 infection. By analyzing paired high-resolution HLA types and viral genomic sequences from 60 patients, we assess the relationship between predicted HLA class I peptide binding repertoires and infection severity as measured by the sequential organ failure assessment score. This approach leverages functional convergence across HLA-C alleles to identify relationships that may otherwise be inaccessible due to allelic diversity and limitations in sample size. Surprisingly, our findings show that severely symptomatic infection in this cohort is associated with disproportionately abundant binding of SARS-CoV-2 structural and non-structural protein epitopes by patient HLA-C molecules. In addition, the extent of overlap between a given patient’s predicted HLA-C and HLA-A peptide binding repertoires correlates with worse prognoses in this cohort. The findings highlight immunologic mechanisms linking HLA-C molecules with the human response to viral pathogens that warrant further investigation.

## 1. Introduction

Severe acute respiratory syndrome coronavirus type 2 (SARS-CoV-2) produces a considerable range of clinical presentations, from mild common cold-like symptoms to severe pneumonia, respiratory distress syndrome (ARDS), and multisystem organ failure (MOF) [1,2]. While patient demographic, medical, and behavioral risk factors certainly influence outcomes [3], dysregulated activation of immune responses is also implicated in severe clinical progression early in infection [4,5].

Human leukocyte antigen (HLA) genes are highly polymorphic structures with central roles in shaping immune responses against pathogens. The interactions between HLA I and II molecules and T cell receptors (TCR) activate lymphocytes and develop the adaptive immune effector milieu. HLA class I antigens interact with cytotoxic T cells (CD8+) and kill virally infected targets. Different HLA genes determine specific repertoires of peptides included in the major histocompatibility complex (MHC) and influence subsequent TCR recognition, leading to antigen-specific immune responses. Therefore, HLA heterogeneity is a crucial factor underlying differential susceptibility to viral infection across human populations.

Distinct HLA alleles and haplotypes impact severity and progression of viral infection due to human immunodeficiency virus (HIV) [6,7,8,9], hepatitis B [10,11], hepatitis C [12,13,14], and influenza [15,16,17]. Certain HLA alleles have also been associated with poor clinical response to infection by SARS-CoV [18,19], a closely related predecessor to SARS-CoV-2 with approximately 80% genetic sequence identity [20,21]. However, geographic restriction of SARS-CoV transmission allowed for examination of only limited pools of HLA alleles. In contrast, the pandemic nature of SARS-CoV-2 has prompted numerous studies analyzing broader ranges of HLA alleles. These efforts have identified multiple associations between patient HLA alleles and disease outcome [22,23,24,25,26,27,28,29,30,31,32]. However, identified associations are not entirely consistent [32,33]. Discrepancies in allele-level associations between studies are at least partially due to geographic variation in HLA genotype frequency. In addition, geographic and temporal SARS-CoV-2 genetic variation further complicates comparison across studies.

SARS-CoV-2 mutational analysis has been a crucial component of vaccine design, and HLA has emerged as an important target [34,35,36,37,38]. *In silico* peptide binding analyses have been instrumental in identifying antigenic SARS-CoV-2 peptides given the particularly large viral peptidome as well as the combinatorial nature of binding groove sequence variation both within and across HLA loci. NetMHCpan is a widely used computational tool that leverages artificial neural networks to predict peptide binding to MHC molecules. This tool, and other tools like it, integrate experimental binding data from multiple MHC alleles and generalize these patterns to predict binding for well-characterized alleles and those with limited experimental data [39]. While high-affinity MHC-peptide binding does not, by itself, equate with immunogenicity, in silico techniques have successfully identified SARS-CoV-2 epitopes capable of eliciting a T cell response, many of which are HLA specific [40,41,42]. Importantly, even sequence variation within SARS-CoV-2 lineages alters viral peptide presentation on HLA molecules [43].

This study investigates the relationship between predicted HLA class I peptide binding repertoires and the clinical response to SARS-CoV-2 infection. By integrating high-resolution HLA typing and SARS-CoV-2 genomic sequencing, we explore associations between viral antigenicity and disease severity at an individual level while controlling for viral genomic variation. Our results highlight an association between HLA-C-mediated viral peptide binding and effective immune responses to SARS-CoV-2 infection that suggests potential avenues for further research into the immunologic mechanisms underlying severe COVID-19.

## 2. Materials and Methods

### 2.1. Patient Selection and Inclusion Criteria

Patients were selected from the University of Michigan COVID-19 Biorepository and were included in the study based on the availability of corresponding high-resolution HLA typing and viral genomic sequencing data. All patients identified were admitted to Michigan Medicine as inpatients due to symptomatic COVID-19 infection between 24 March 2020 and 5 May 2020. Patients admitted for other comorbidities during initial SARS-CoV-2 testing and those with previous positive SARS-CoV-2 testing outside of Michigan Medicine were excluded to ensure uniformity in baseline characteristics. The study was approved by the University of Michigan Institutional Review Board (HUM00183348).

### 2.2. Assessment of Clinical Response to Infection

Infection severity was evaluated using the Sequential Organ Failure Assessment (SOFA) score, a widely recognized tool for assessing organ dysfunction in critically ill patients [44]. SOFA scores were calculated for each patient every day, and the required parameters were available over 10 days following initial positive SARS-CoV-2 testing. In subsequent analyses, the maximum SOFA score recorded during these 10 days was used as the primary measure of infection severity.

### 2.3. High-Resolution Human Leukocyte Antigen-Typing

Blood collection and subsequent DNA isolation were performed by the University of Michigan COVID-19 Biorepository. After extraction, DNA concentration and quality were measured with the Qiagen Qiexpert spectrophotometer. Next-generation sequencing (NGS) human leukocyte antigen (HLA)-typing for HLA-A, -B, and -C was done using NGSgo^®^-Amp v2 (GenDx, Utrecht, Netherlands), according to the manufacturer’s instructions. After long-range PCR amplification of each HLA gene, DNA fragments were selected, amplified, cleaned, and sequenced on a MiSeq using MiSeq Reagent Kits v2 (300 cycles) (Illumina, San Diego, CA, USA). Samples were analyzed using NGSengine v. 2.25.0 software. Samples were obtained from the University of Michigan Medical School Central Biorepository.

### 2.4. Peptide Binding Predictions

Translated SARS-CoV-2 protein sequences corresponding to viral strains sequenced in this study were obtained from the GISAID database (https://gisaid.org/EPI_SET_220330me accessed on 21 August 2021) [45] and are listed in Table S1. FASTA formatted sequences were parsed using the Bio.Seq module implemented in Biopython (v. 1.75). For each viral strain, protein sequences were partitioned into libraries of all possible overlapping 8–11 amino acid peptides, respectively, using a sliding window method. Binding predictions were then carried out for each patient’s MHC protein toward the corresponding peptide library using local installations of NetMHCpan (v. 4.1) artificial neural networks [39] with default input parameters. Binding affinity (BA) and mass-spectrometry eluted ligand (EL) data were used to select predicted interactions for further analysis. Peptide binding data in comma-separated values (CSV) format was read and merged with patient sample information using pandas (v. 1.4.3). NumPy (v. 1.21.3) was utilized for performing arithmetic operations, such as logarithmic transformations and normalization. Predicted BA and EL results for 2431 experimentally examined MHCI:peptide pairs available in the IEDB were used to generate a logistic regression model, and the resulting probabilities were evaluated by ROC analysis using the LogisticRegression and roc_curve functions available in the scikit-learn (v. 1.3.2) Python library.

### 2.5. Protein Sequence Analysis, Modeling, and Visualization

Aligned HLA-C amino acid sequences were obtained from the IPD-IMGT/HLA Database [46] in FASTA format. Three-dimensional structures were generated for each patient HLA-C protein using the ColabFold [47] implementation of AlphaFold2 [48]. Electrostatic surface potentials were calculated and visualized using the Adaptive Poisson–Boltzman Solver (APBS) plugin for PyMOL (v. 2.5). Surface potentials are represented as a color gradient from red (−5 $k_{b}$*T*/$e_{c}$) to white (0 $k_{b}$*T*/$e_{c}$) to blue (5 $k_{b}$*T*/$e_{c}$), where $k_{b}$ is the Boltzmann constant, *T* is the absolute temperature in Kelvin, and $e_{c}$ is the elementary charge. The model of HLA-C*03:04 bound to a SARS-CoV-2 NSP3 peptide (residues 1885–1894, UniProt ID: P0DTC1) was generated using AlphaFold-Multimer (https://doi.org/10.1101/2021.10.04.463034) followed by refinement using Rosetta FlexPepDock low- and high-resolution protocols (20455260). All protein visualizations were prepared using PyMOL (v. 2.5), and peptide sequence logos were generated using the Logomaker library implemented in Python (v. 3.8). For sequence-based clustering analysis, residues at positions forming the HLA-C peptide binding surface were embedded using sgt (v. 2.0.3), a Python implementation of sequence graph transform. PCA and k-means clustering analysis was then performed on embedded sequences using the scikit-learn library (v. 0.24.2) in Python (v. 3.8). PCA scatter plots were prepared using the Matplotlib library (v. 3.4.2) implemented in Python (v. 3.8).

### 2.6. Statistical Analysis

Bayesian generalized linear multivariate regression was performed using the brms implementation of Stan in R [49,50] using the following equation:

(1) $$y_{i}=\beta_{0}+\beta_{1}A_{i}^{*}+\beta_{2}B_{i}^{*}+\beta_{3}C_{i}^{*}+\beta_{4}{\mathrm{Sex}}_{i}+\beta_{5}{\mathrm{Age}}_{i}+\beta_{6}{\mathrm{BMI}}_{i}+\epsilon_{i},$$

where $i_{i}$ refers to the SOFA score corresponding to the *i*-th patient, $\beta_{0}$, $\beta_{1}$,…, $\beta_{6}$ are the estimated coefficients, and $\epsilon_{i}$ is the error term. $A_{i}^{*}$, $B_{i}^{*}$, and $C_{i}^{*}$ are the latent true values of the HLA-A, -B, and -C peptide repertoire sizes calculated across binding probability thresholds 0.5 to 0.95, accounting for error using the me(mean, standard deviation) brm function call. Sex, age, and BMI are additional predictors without associated measurement errors. All predictors were z-score normalized before multivariate regression. Confidence intervals were calculated via bootstrapping with 100 resamples, using the boot function implemented in R. The relationship between SOFA score and the fractional intersection of patient HLA-A (or -B) and HLA-C peptide binding repertoires was similarly modeled using the equation:

(2) $$y_{i}=\beta_{0}+\beta_{1}X_{i}^{*}+\epsilon_{i},$$

with $y_{i}$, $\beta_{0}$, $\beta_{1}$, and $\epsilon_{i}$ defined as in Equation (1) and $X_{i}^{*}$ referring to the intersection of the *i*-th patient’s HLA-A (or -B) and HLA-C peptide binding repertoires divided by HLA-A (or B) repertoire size. Model visualizations were prepared using the Matplotlib (v. 3.4.2) and Seaborn (v. 0.11.2) Python libraries.

Pearson correlations were calculated using the pearsonr method implemented in the stats module in the SciPy Python library (v. 1.6.2). Analysis of variance (ANOVA) and *t*-test calculations were performed using the SciPy f_oneway and ttest_ind methods, respectively. A *p*-value cutoff of 0.05 was used to identify statistically significant results in all analyses described above. Data visualizations were prepared using the Matplotlib (v. 3.4.2) and Seaborn (v. 0.11.2) Python libraries.

## 3. Results

### 3.1. Patient Cohort Characteristics

**Figure 1:**
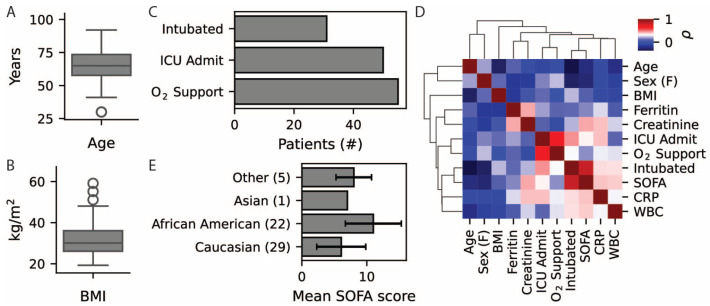
Patient cohort characteristics. (**A**,**B**) Box and whisker plots showing age and BMI distributions across the cohort. (**C**) Bar plot showing the number of patients that required supplemental oxygen support, intensive care unit (ICU) admission, and intubation. (**D**) Correlation map showing relationships between cohort characteristics and outcome measures. (**E**) Bar plot showing mean SOFA scores for patients based on self-reported ancestry (SRA). Error bars represent standard deviation from the mean.

The study includes 60 patients consisting of 38 men and 22 women aged 65 ± 14 years (Figure 1A) with body mass index (BMI) 32 ± 9 kg/m^2^ (Figure 1B). Of these patients, 55 required supplemental oxygen support, 50 were admitted to the ICU, and 31 required intubation (Figure 1C). Subsequent statistical analyses in this study quantify infection severity as the maximum sequential organ failure assessment (SOFA) score documented within 10 days of SARS-CoV-2 diagnosis. In this cohort, SOFA score is negatively correlated with age (*p* = 0.06) and female sex (*p* = 0.04) and positively correlated with ICU admission (*p* = 0.009) and intubation requirement (*p* = $3\times 10^{-15}$, Figure 1D). Supplemental oxygen requirement was the only clinical management outcome that did not significantly correlate with SOFA score (*p* = 0.19), as all but five patients required this intervention. Notably, patients of African-American self-reported ancestry (SRA) demonstrated higher SOFA scores (11.0 ± 4.3) compared to patients reporting Caucasian, Asian, or other SRA (6.1 ± 3.8, 7.0, and 8.0 ± 2.7, Figure 1E).

### 3.2. Extent of Patient HLA-C Peptide Binding Repertoire Is a Risk Factor for Severe SARS-CoV-2 Infection

**Figure 2:**
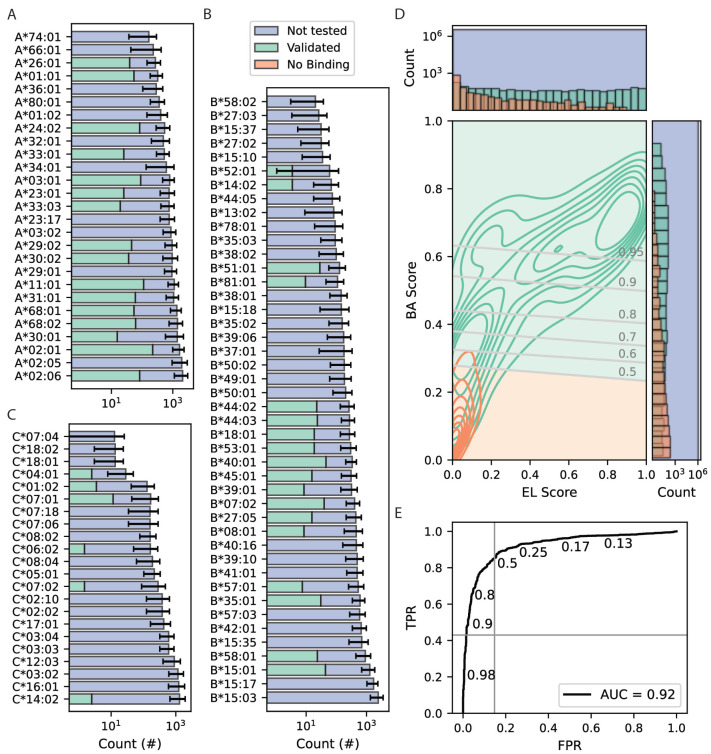
Assessment of peptide binding predictions. (**A**–**C**) Total number of SARS-CoV-2 peptides predicted to bind each MHC molecule (purple bars). Error bars represent standard deviation across binding probability thresholds of 0.5 to 0.95 at intervals of 0.01. Subsets of interactions documented in the IEDB are shown as green bars. (**D**) Contour plot and bar plots showing distributions of netMHCpan-4.1 BA and EL scores for predicted interactions. Green and orange lines/bars correspond to interactions showing positive and negative binding in the IEDB, respectively. Purple bars show the total number of predicted binding interactions. Gray lines indicate probability thresholds for positive binding. (**E**) ROC analysis of a logistic regression probability model for identifying positive binding predictions. The vertical and horizontal gray lines denote the 0.5 and 0.95 binding probability thresholds, respectively.

To allow associations between the extent of viral peptide binding by patient-encoded MHC class I molecules and clinical outcomes following SARS-CoV-2 infection, binding predictions were performed using NetMHCpan-4.1 software. Prior studies indicate that binding interactions with %Rank scores < 20% correspond with positive predictive values (PPVs) > 80% [39]. Binding affinity (BA) and mass spectrometry-based eluted ligand (EL) scores were calculated for each patient’s three pairs of MHC class I molecules toward all possible 8- to 11-mer peptides derived from their SARS-CoV-2 peptidome (Figure 2A–C, Tables S2–S4). In addition, these MHCI:peptide pairs were queried in the Immune Epitope Database (IEDB) to identify experimentally validated binding interactions and pairs showing no binding. The resulting 2431 MHCI:peptide pairs were used as a training set for logistic regression, including binding and non-binding MHCI:peptide pairs with equal weights. The resulting model combines the separate BA and EL scores into a composite binding probability, which is most closely related to the BA score (Figure 2D). The receiver operating characteristic analysis of the logistic regression model shows an optimal balance of sensitivity and specificity at a binding probability threshold of 0.5 (Figure 2E). However, maximizing specificity may also be desirable to emphasize peptides with high predicted affinity. Since a specific binding affinity cutoff within this range is unclear, all binding probability thresholds between 0.5 and 0.95 (Figure 2D,E) in increments of 0.01 were assessed in downstream statistical analyses to avoid spurious results that may stem from a single arbitrary selection.

**Figure 3:**
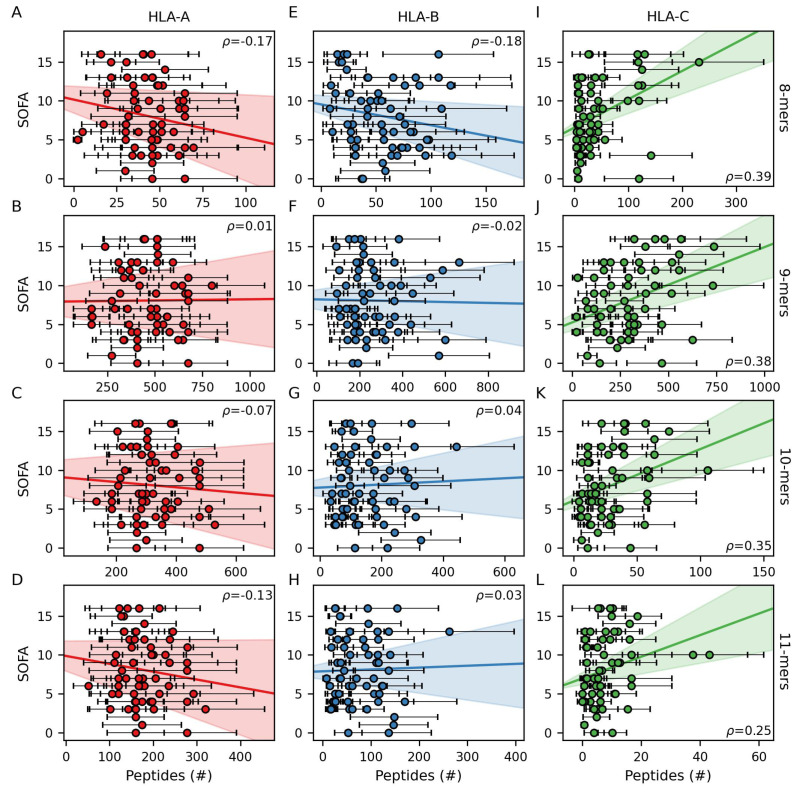
Association between infection severity and HLA class I peptide repertoire size. Correlations between peptide repertoire size (stratified by peptide length) predicted for each patient and SOFA scores are shown for HLA-A (**A**–**D**), -B (**E**–**H**), and -C (**I**–**L**). Straight lines and shading represent linear regression fit and 95% confidence intervals, respectively. Error bars represent standard deviation across binding probability thresholds of 0.5 to 0.95 at intervals of 0.01.

**Figure 4:**
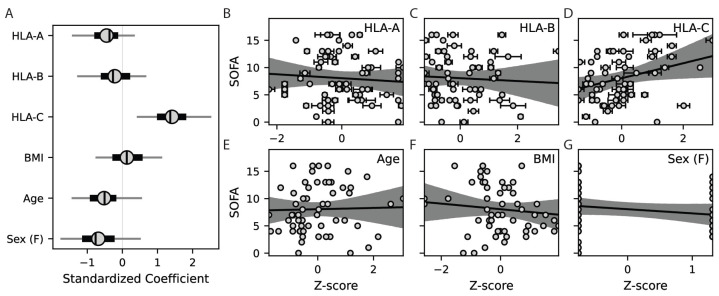
Total number of predicted HLA-C/SARS-CoV-2 peptide interactions predict infection severity. (**A**) Forest plot showing standardized multiple linear regression coefficients (circles) for predictors of patient SOFA scores, including 50% (thick lines) and 95% (thin lines) confidence intervals. Scatter plots show the contributions of the z-scored individual regressors to the overall model, including (**B**–**D**) HLA-A, -B, and -C peptide binding repertoire sizes predicted for each patient, as well as patient (**E**) age, (**F**) BMI, and (**G**) sex. Error bars represent standard deviations of the independent variables across binding probability thresholds of 0.5 to 0.95 at intervals of 0.01, and shading represents the 95% confidence interval of the linear fit.

To test the hypothesis that response to infection is related to the total sizes of patient HLA class I peptide binding repertoires, we assessed correlations between SOFA score and the sum of predicted binding interactions for each patient’s pair of HLA-A, -B, and -C alleles across 8- to 11-mers (Figure 3A–L). These analyses show that patient SOFA scores are positively correlated with the total number of peptides predicted to bind their HLA-C molecules (*p* = 0.002 to 0.05 depending on peptide length, Figure 3I–L) but not necessarily their HLA-A or -B molecules (*p* = 0.19 to 0.94 and *p* = 0.17 to 0.88, respectively, Figure 3A–H). We then used the summed binding interactions across 8- to 11-mers for each patient’s two HLA-A, -B, and -C alleles to predict SOFA score through a Bayesian generalized linear multivariate regression framework, adjusting for patient age, BMI, and sex (Figure 4A–G).

In attempting to control for SRA as a covariate in the model, we identified very strong collinearity between self-reported ancestry and HLA-B allele status in this cohort, with variance inflation factor (VIF) = 13.81 and the corresponding R^2^ = 0.93. Such strong collinearity threatens the validity of inferences drawn from a general linear model due to the risk of introducing numerical instability to the parameter estimates [51]. As a result, we judged that, while both variables could not be included in the model, either variable represents self-reported ancestry with adequate accuracy. However, of the two variables, only HLA-B allele status allows examination of linkage disequilibrium, local (rather than global) ancestry estimation, and conditional effects. As a result, we elected to allow HLA-B allele status to address possible confounds flowing from HLA-independent inequality factors and HLA-independent ancestry-related genetic effects in the model.

To avoid spurious results due to ambiguity regarding biologically relevant MHCI:peptide binding affinities and using a single arbitrary cutoff to define binding, numbers of peptide interactions were calculated over a range of binding probability cutoffs (0.5–0.95, Figure 2E) at intervals of 0.01, and the resulting distributions were accounted for as independent variable uncertainty using Bayesian techniques. To test the stability and reliability of the estimated coefficients, a bootstrap method was employed with 100 resamples, and the original coefficients, bootstrapped coefficients, and bootstrapped confidence intervals are shown as a forest plot (Figure 4A). Of the variables examined, increased size of HLA-C peptide binding repertoire predicts poor response to infection (coefficient = 1.41 ± 0.58, Figure 4A,D). Although not reaching statistical significance, increases in SOFA score tended to be associated with decreases in the size of HLA-A (coefficient = −0.44 ± 0.47) and, to a lesser extent, -B (coefficient = −0.22 ± 0.58) peptide binding repertoire sizes. Of note, HLA class I molecules are known to be highly biased toward 9-mer peptides, which is also observed in this predictive analysis (Figure 3B,F,J). Explicitly including this bias by repeating the analysis with only the 9-mer predictions yielded similar findings (HLA-A, -B, and -C coefficients = −0.32 ± 0.59, −0.24 ± 0.60, and 1.33 ± 0.65, respectively). These results show that disproportionate peptide binding by HLA-C molecules relative to HLA-A and -B is a risk factor for severe SARS-CoV-2 infection in this cohort of patients.

### 3.3. Disproportionate HLA-C Binding of Structural and Nonstructural Protein Sequences Contributes to Poor Prognosis

**Figure 5:**
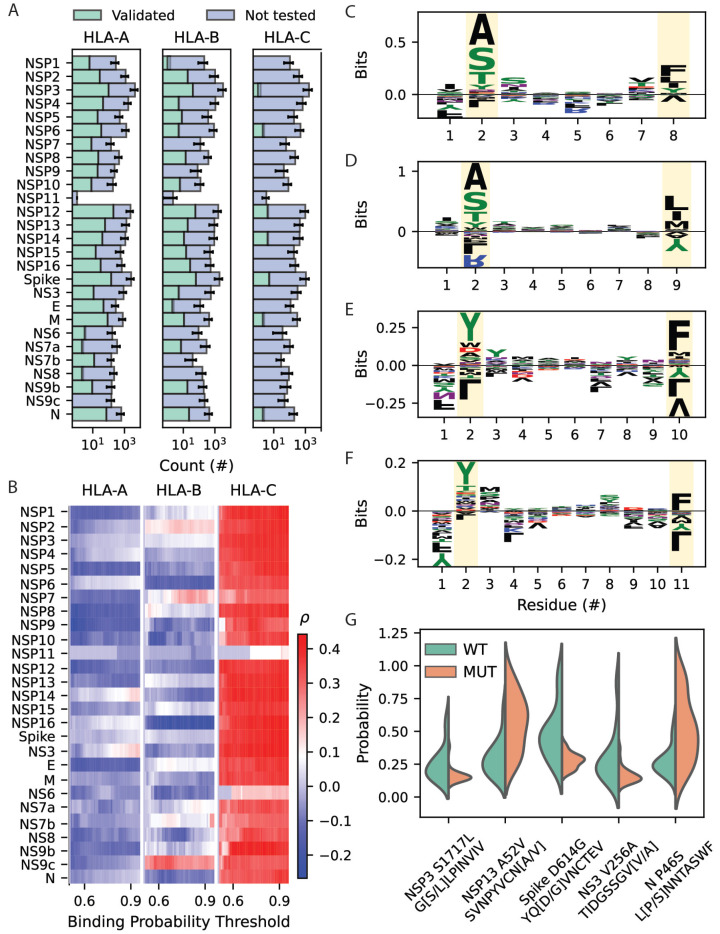
Severity of illness correlates with predicted HLA-C-mediated binding of structural and non-structural SARS-CoV-2 protein sequences. (**A**) Distribution of predicted MHC class I binding across the SARS-CoV-2 proteome. (**B**) Heatmaps showing a correlation between predicted number of binding interactions and SOFA score across SARS-CoV-2 proteins. (**C**–**F**) Sequence enrichment plots for 8 to 11 amino acid peptides exhibiting the greatest magnitude correlation (top 5%) between HLA-C binding probability and SOFA score. (**G**) Split violin plots showing effects of missense mutations on predicted binding probability toward patient HLA-C molecules. Binding probability distributions for wild-type (Wuhan strain) and mutant strains are shown in green on the left and orange on the right, respectively. All distributions shown significantly differ between wild-type and mutant peptides (*p* < 0.05).

Early efforts aimed at epitope mapping primarily focused on SARS-CoV-2 structural proteins, particularly spike proteins. However, peptides derived from nonstructural and accessory proteins can also elicit an immune response to SARS-CoV-2 infection [52,53]. As a result, we examine the HLA class I binding of sequences derived from all 27 SARS-CoV-2-encoded proteins (Figure 5A). To identify protein sequences leading to unbalanced HLA-C peptide binding relative to HLA-A and -B, predicted repertoire sizes for individual SARS-CoV-2 proteins were compared with the SOFA score for each patient. HLA class I peptide repertoire sizes for each SARS-CoV-2 protein in aggregate were calculated across binding probability thresholds 0.5 to 0.95 at intervals of 0.01. Significant relationships with SOFA score were then identified with Pearson correlations corresponding to *p* < 0.05. In this analysis, quantities of predicted HLA-C peptide-binding interactions show a significant positive correlation with SOFA score for 20 of the 27 SARS-CoV-2 proteins (Figure 5B). Conversely, peptide repertoire sizes for HLA-A and -B are predominantly lower for patients with more severe infection, although these correlations for peptidomes derived from individual protein sequences do not reach statistical significance. These results indicate that the disproportionate binding of HLA-C molecules toward peptides derived from structural, nonstructural, and accessory SARS-CoV-2 protein sequences contributes to an elevated risk of severe infection.

Peptide sequence characteristics associated with differential HLA-C binding in mild versus severe SARS-CoV-2 infection were also examined. Pearson correlations were calculated between HLA-C binding probability and SOFA score for all peptides, and the top 5% of positively and negatively correlated sequences were compared. The resulting sequence enrichment plots show residues that contribute most to the correlation between SOFA score and predicted peptide binding of each patient’s HLA-C alleles (Figure 5C–F). Peptide sequences predicted to bind to HLA-C alleles in patients with severe SARS-CoV-2 infection show a preference for amino acid restriction at the second from *N*-terminal (P2) and *C*-terminal anchor positions. The *C*-terminal position is enriched for hydrophobic residues, which is consistent with HLA-C binding specificity overall [54]. The 8- and 9-mer peptides show P2 enrichment for Ser, Thr, and Ala in peptides differentially bound by HLA-C alleles encoded by patients with severe versus mild infection (Figure 5C–F). Subsets of these P2 residues have been shown to be preferred by HLA-C*02, -C*03, -C*05, -C*08, -C*12, -C*15, and -C*16 molecules [54]. Surprisingly, P2 enrichment shifts toward Tyr for the longer 10- and 11-mer peptides, which is preferred by C*04:01, C*06:02, and C*07:02. Once again, though, it is important to note that HLA class I molecules are highly biased toward binding 9-mer peptides [55], as demonstrated by the netMHCpan-4.1 predictions (Figure 3).

While all SARS-CoV-2 sequences identified in this study are of either the A or B.1 lineage (Table S1), amino acid differences exist at 62 missense mutation sites. Five of these mutations significantly alter the distributions of binding probability scores across patient HLA-C alleles (Figure 5G). Mutations of non-structural protein 3 (NSP3) and nucleocapsid protein (N) affect the P2 anchor, where Ser is favored over Gly and Pro. Mutations involving the *C*-terminal anchor of peptides derived from NSP13 and the NS3 accessory protein highlight preference for Val over Ala at this position. In addition, the Spike Asp^614^Gly mutation associated with enhanced viral fitness is predicted to result in less HLA-C binding in this patient cohort (Figure 5G). These results implicate structural, nonstructural, and accessory protein sequences in differential HLA-C peptide binding and prognostic impact. Furthermore, missense mutations within single SARS-CoV-2 lineages may be expected to alter binding by HLA-C molecules due to single amino acid changes.

### 3.4. Infection Severity Positively Correlates with Overlap between HLA-A and -C Peptide Binding Repertoires

**Figure 6:**
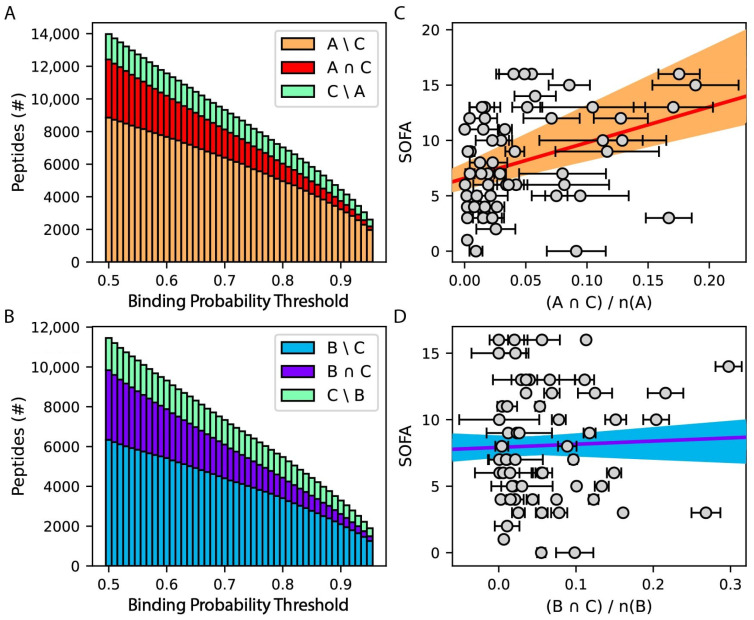
Infection severity correlates with the extent of overlap between SARS-CoV-2 peptide repertoires for patient HLA-A and -C molecules. (**A**,**B**) Stacked bar chart showing the intersection and differences between sets of SARS-CoV-2 peptides predicted to bind each patient’s HLA-A, -B, and -C molecules. (**C**,**D**) Results of linear regression using fractional intersection of peptide sets predicted to bind each patient’s HLA-A/B and -C molecules to predict SOFA score. Error bars represent standard deviation across binding probability thresholds of 0.5 to 0.95 at intervals of 0.01.

Unbalanced viral peptide binding by HLA-C relative to HLA-A and -B during infection may be exacerbated by competition for shared ligands. Indeed, distributions of predicted peptide binding across SARS-CoV-2 proteins are similar for each of the three HLA class I loci (Figure 5A). To test this hypothesis, the intersection between sets of SARS-CoV-2 peptides recognized by each patient’s HLA-A, -B, and -C alleles was determined over a range of prediction scoring thresholds (Figure 6A,B). For all patient alleles combined, percentages of HLA-A and -B peptide repertoires also predicted to be bound by HLA-C alleles range from 10–29% and 16–36%, respectively (Figure 6A,B). The intersection between HLA-A and -C peptide repertoires significantly correlates with SOFA score (Figure 6C, *p* < 0.004), but a significant relationship between HLA-B and -C peptide repertoires is not identified (Figure 6D). We also investigated how this overlap may contribute to our multivariate linear regression model (Figure 4). Repeat regression analysis after removing peptides shared between HLA-C and -A or -B incrementally strengthens the positive association between HLA-C peptide binding and SOFA score by 1.05-fold. Furthermore, removing peptides predicted to bind HLA-C potentiates the negative correlation between HLA-A and -B peptide binding and SOFA score by 1.14- and 1.46-fold, respectively. These results suggest that competition between HLA-A and -C for shared SARS-CoV-2 epitopes contributes to inefficient HLA class I responses, predisposing patients to severe infection. HLA-C competition for viral peptide loading in the endoplasmic reticulum may lead to attenuated antigen presentation due to less efficient trafficking to the cell surface, as HLA-C requires higher local peptide concentrations to form stable peptide complexes [56]. Indeed, the fractional overlap between HLA-C and -A/-B peptide repertoires is inversely correlated with predicted binding probability (Figure 6A,B), suggesting this competition has the greatest impact on peptides involved in low-affinity interactions. HLA-C alleles expressed at higher levels may be expected to overcome this mechanism. However, we do not observe a correlation between HLA-C surface expression [57] and SARS-CoV-2 infection severity in our cohort. An explanation for this discrepancy may be that the substantial difference between HLA-C and HLA-A/B expression levels obscures the effects of smaller differences among HLA-C expression levels in this analysis. In addition, an alternative hypothesis is that peptide repertoire overlap between HLA class I loci reduces the functional diversity of antigen presentation, which has recently been associated with more rapid disease progression in HIV infection [58]. In either case, the findings warrant further investigation into how competition between HLA class I molecules affects the immune response to viral infection.

### 3.5. HLA-C1 KIR Ligand Group Alleles Are Predicted to Bind Larger Numbers of Peptides and Are Associated with Increased Infection Severity

**Figure 7:**
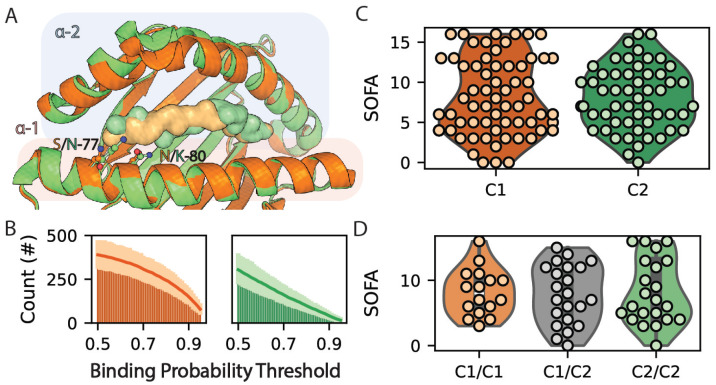
Relationship between KIR cluster, predicted SARS-CoV-2 peptide binding, and illness severity. (**A**) Structural overlay of C1 (HLA-C*07:02; PDB ID 5VGE) and C2 (HLA-C*06:02; 5W6A) molecules. (**B**) Mean numbers of predicted SARS-CoV-2 peptide-binding interactions for C1 and C2 molecules as a function of binding probability threshold. (**C**) Distributions of patient SOFA scores corresponding to individual C1 and C2 alleles. (**D**) Dependency of patient SOFA score on patient C1/C2 genotype).

HLA-C molecules are the primary ligands of killer cell immunoglobulin-like receptors (KIRs), thus playing an important role in natural killer (NK) cell-mediated responses to infection [59,60]. A balanced dimorphism at amino acids 77 and 80 divides HLA-C molecules into C1 and C2 KIR ligand groups [61] (Figure 7A). C1 allotypes have previously been predicted to bind larger quantities of SARS-CoV-2 peptides [62], and several have been individually identified as risk factors for poor prognosis [22,25,28,63,64,65,66,67]. In the present study, C1 allotypes are predicted to bind larger numbers of SARS-CoV-2 peptides than C2 allotypes (*p* < 0.002, Figure 7B). While the KIR ligand group for single HLA-C alleles is not correlated with SOFA scores (Figure 7C, *p*> 0.98), a significant dose-dependent correlation exists between patient HLA-C1/C2 genotype and SOFA score, where increasing C1 allotypes in a given patient are associated with more severe infections (ANOVA *p*< 0.04, Figure 7D). This finding again links higher HLA-C peptide binding capacity with severe infections. Future studies will be needed to investigate the impact of increased viral antigen presentation by HLA-C1 versus C2 allotypes on KIR interactions and the NK cell response.

### 3.6. The Amino Acid Sequence of the HLA-C Peptide Binding Groove Predicts Patient Prognosis Following SARS-CoV-2 Infection

**Figure 8:**
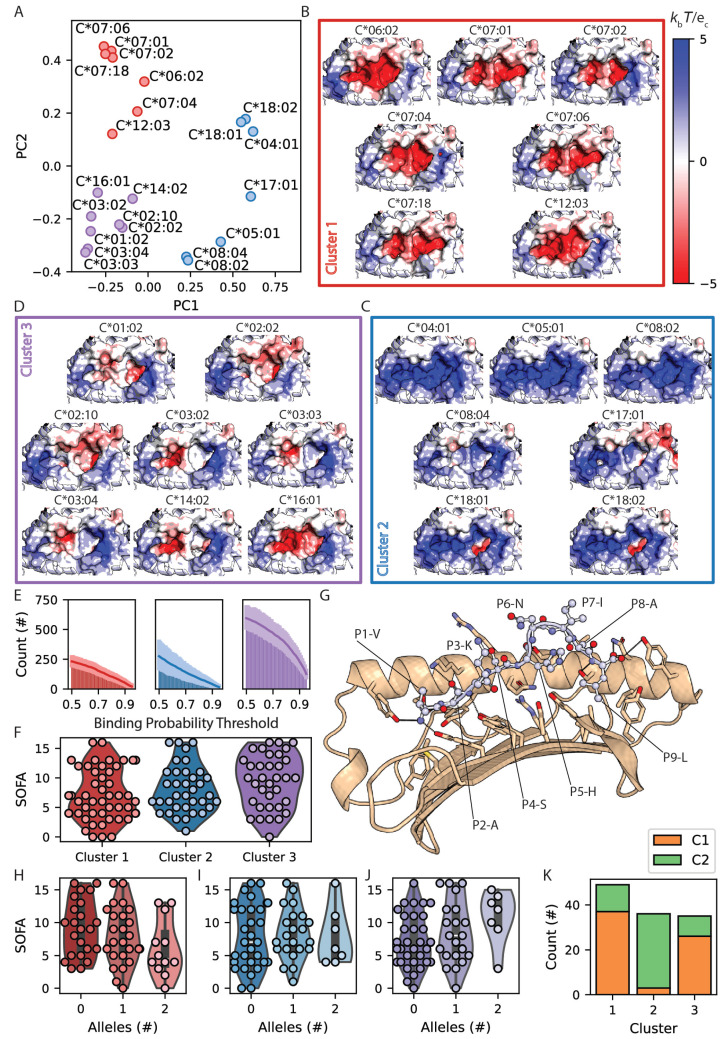
Effect of HLA-C peptide binding surface amino acid composition on predicted peptide repertoire and infection severity. (**A**) PCA and k-means clustering of patient HLA-C amino acid sequences. (**B**–**D**) Electrostatic potential at the HLA-C peptide binding grooves is represented as a color gradient from red (−5 $k_{b}$*T*/$e_{c}$) to white (0 $k_{b}$*T*/$e_{c}$) to blue (5 $k_{b}$*T*/$e_{c}$) and grouped by sequence-based cluster. (**E**) Mean numbers of predicted SARS-CoV-2 peptide-binding interactions for each cluster across binding probability thresholds. (**F**) Distribution of patient SOFA scores for individual alleles in each cluster. (**G**) Docked structure of HLA-C*03:04 bound to a 9 amino acid SARS-CoV-2 NSP3 peptide (residues 1885–1894, UniProt ID: P0DTC1). (**H**–**J**) Dose dependence of patient SOFA score on genotypic composition for allele clusters 1, 2, and 3 in each patient. (**K**) C1/C2 status composition of alleles in each cluster.

The balanced dimorphism defining KIR ligand groups has a limited impact on the HLA-C peptide binding surface (Figure 7A). To comprehensively examine the relationship between HLA-C amino acid sequence and differences in patient prognoses, alleles were clustered based on their complete peptide binding site amino acid sequences (Figure 8A). Distinct electrostatic patterns between the resulting groups delineate Cluster 1 and 2 peptide binding grooves as predominantly negatively and positively charged, respectively, while Cluster 3 binding sites are relatively hydrophobic (Figure 8B–D). Comparing patient SOFA scores with each of their two HLA-C alleles reveals differential prognosis across sequence-based clusters (Figure 8E, ANOVA *p* = 0.05). This analysis, too, demonstrates that severe SARS-CoV-2 infection is associated with larger HLA-C peptide binding repertoires (Figure 8F, ANOVA *p* <$1\times 10^{-11}$). Cluster 3 alleles are predicted to have the largest peptide binding repertoire and are associated with the greatest prognostic risk. Cluster 3 peptide binding promiscuity may be associated with the preference of their relatively hydrophobic binding pockets to bind peptides with uncharged residues at P2 (8 G), which are correlated with patient SOFA scores (Figure 5C–F). Conversely, Cluster 1 is associated with better prognoses and is mostly composed of HLA-C*06 and -C*07 alleles, which prefer Arg at P2 [54], consistent with their net negatively charged peptide-binding surfaces (Figure 8B). Clusters 1 and 3 showed no significant allele dosage effects with respect to SOFA scores (ANOVA *p* = 0.15 and 0.12, respectively; Figure 8H,J). However, patients with two Cluster 3 alleles demonstrated significantly higher SOFA scores than patients with zero Cluster 3 alleles (*t*-test *p* = 0.03, Figure 8J). Cluster 2 is associated with intermediate prognosis (Figure 8F) and also does not show an allele dosage effect (ANOVA *p* = 0.96, Figure 8I). Variation in HLA-C surfaces likely affects KIR interactions in addition to peptide binding. While Cluster 2 is composed almost exclusively of HLA-C2 KIR ligand group alleles (92%), the divergent Clusters 1 and 3 each comprise mostly HLA-C1 group alleles (76 and 74% respectively, Figure 8K). This similarity is unexpected since the number of HLA-C1 KIR ligand group alleles for a given patient genotype are overall associated with higher SOFA scores (Figure 7D). These results support a mechanism by which HLA-C molecule amino acid composition and differential peptide binding specificity impact patient response to SARS-CoV-2 infection that is non-redundant with KIR ligand specificity, and future studies are needed to investigate these mechanisms.

## 4. Discussion

The HLA system is a crucial determinant of infection susceptibility, making it an attractive target for investigating genetic causes of differential responses to pathogens across human populations. HLA class I molecules are important for the early elimination of virus-infected cells by CD8+ T cells and NK cells while maintaining self-tolerance to avoid excessive tissue damage. As a result, HLA class I genotypes have received significant attention with respect to aberrant immune responses during early SARS-CoV-2 infection. Here, we compare HLA class I genotypes and strain-specific SARS-CoV-2 peptidomes for 60 patients seen at Michigan Medicine early in the COVID-19 pandemic to investigate relationships between predicted peptide binding and infection severity. This analysis shows patients with larger quantities of HLA-C/viral peptide interactions were significantly more likely to experience severe symptoms (Figure 3 and Figure 4).

HLA-C molecules are expressed in lower quantities at the cell surface [68,69,70,71], compared with HLA-A and -B and, therefore, often receive less attention in studies of responses to viral infection. Nevertheless, HLA-C-restricted viral peptides have been implicated during disease progression in HIV [72,73,74,75,76,77] and hepatitis C infections [78]. While the role of HLA-C in viral respiratory illness remains poorly understood, specific HLA-C alleles are associated with differential risk of infection and/or prognosis in influenza [79,80,81], chikungunya [82], dengue [83], lassa [84], and ebola [85,86,87] viruses. Increasing evidence also links genetic and epigenetic polymorphism at the HLA-C locus with differential clinical outcomes in SARS-CoV-2 infection. Epigenome-wide association studies implicate differential CpG methylation of the HLA-C locus in conferring risk for developing severe infection with respiratory failure [88]. In addition, multiple studies have identified associations between specific HLA-C alleles and prognosis following SARS-CoV-2 infection [29,30,31,89,90,91], albeit with at times conflicting results [32,33]. Potential causes for this variability include differences in the populations and/or SARS-CoV-2 strains examined and difficulty obtaining large sample sizes. Our study addresses statistical limitations associated with these issues by assessing predicted viral peptide binding as a continuous variable rather than discrete HLA alleles. In addition, predicting peptide binding with respect to each patient’s sequenced SARS-CoV-2 strain controls for differences in viral peptidomes and would allow for unbiased comparison with other cohorts.

Previous studies also suggest an association between poor clinical outcome and HLA-A and -B allotypes with poor capacity for viral peptide binding [23,25]. We hypothesize that HLA-C molecules may exacerbate this problem by competing with other HLA class I molecules for peptide binding, potentially causing lower antigenicity due to less efficient cell surface presentation by HLA-C versus -A and -B molecules. Future studies could test this hypothesis by quantifying peptides presented by HLA-A and -B in cells encoding HLA-C alleles with varying degrees of expected overlap in peptide binding repertoire. In addition, overlap in peptide binding repertoires between HLA class I loci may attenuate functional diversity, which was recently associated with more rapid disease progression due to HIV infection [58]. Indeed, we show higher degrees of overlap between predicted HLA-A and -C molecule peptide binding specificity for individual patients positively correlate with infection severity (Figure 6C).

Prior studies have identified roles for HLA-C/KIR relationships in differential responses to SARS-CoV-2 infection [89,90,91,92]. HLA-C1 allotypes are predicted to bind larger quantities of SARS-CoV-2 peptides than HLA-C2 [62] (Figure 7B). HLA-C1 allotypes also show a dose-dependent correlation with SOFA score in our cohort (Figure 7D), consistent with the hypothesis that promiscuity in HLA-C peptide binding contributes to infection severity. This finding highlights the need to further investigate the impact of HLA-C1/C2 classification on T cell recognition, as there has only been one published study on this subject to date [93]. We also orthogonally classified HLA-C alleles based on the complete peptide binding groove amino acid composition and found that clusters with higher promiscuity in peptide binding were associated with more severe infection (Figure 8). Here, the two clusters associated with the best and worst prognoses are composed mostly of HLA-C1 allotypes, whereas the intermediate cluster includes almost exclusively HLA-C2 allotypes. These findings support non-redundant roles for HLA-C peptide binding capacity and KIR interactions in tuning immune responses to SARS-CoV-2 infection.

### Limitations of the Study

While our inclusion/exclusion criteria required that patients were admitted to our institution as inpatients due to new symptomatic SARS-CoV-2 infection as opposed to acquiring infection while hospitalized for another comorbidity, we have relatively little information about the cohort baseline health status as a whole since many of the patients had not been seen in our system recently before presentation.

In addition, the uncertain functional significance of in silico peptide-binding prediction tools represents another inherent limitation to the present study. This limitation is partly addressed by using available experimental data from the IEDB to refine initial predictions; however, there are relatively few experimental results for the HLA-C/SARS-CoV-2 peptide pairs (Figure 2). Furthermore, MHC:peptide binding does not guarantee efficient antigen presentation or subsequent T cell response. As a result, future studies would be needed to confirm binding predictions and investigate functional relationships with T cell activation.

Another fundamental limitation of our study is the decision to use HLA-B allele status to control for both global ancestry and local ancestry. In particular, since SOFA score was selected as the dependent variable in this study, the need to account for confounds flowing either from non-HLA genetic effects or ancestry-related social inequality factors [94] is crucial to the soundness of our findings. The most direct limitation of our approach is that HLA-B status is a robust predictor of, but not a perfect proxy for, global ancestry in our cohort. As such, influences of HLA-independent factors associated with ethnicity cannot be entirely excluded. Future research studies in this area could consider a stratified study design to eliminate this confound cost-effectively. Alternatively, a more extensive study could tease independent effects apart without stratification.

Although pairing high-resolution HLA-typing with viral sequencing to control for patient-specific viral peptidomes in peptide binding predictions represents a strength of the present study, this imposes a significant related limitation in that relatively few samples (n = 60) were available for analysis. This limitation highlights the need for replication cohorts with paired HLA-typing and viral sequencing to validate the findings further.

## 5. Conclusions

This study identifies a link between HLA-C peptide binding repertoires and the severity of SARS-CoV-2 infections observed at Michigan Medicine in the early stages of the COVID-19 pandemic. Patients with more extensive predicted HLA-C-mediated viral peptide repertoires experienced more severe symptoms, as reflected in higher SOFA scores. Additionally, the extent of overlap between HLA-C and HLA-A peptide binding correlated with increased infection severity, suggesting competition for shared peptides may contribute to poor immune response. These findings highlight a perhaps underappreciated role for HLA-C in shaping immune responses to SARS-CoV-2 infection that warrants further investigation.

## Data Availability

The findings of this study are based on 60 sequences available on GISAID between 27 March 2020 and 9 May 2020, accessible at https://gisaid.org/EPI_SET_220330me accessed on 8 August 2021. All code used for downstream analysis is publicly available at the corresponding author’s GitHub profile at https://github.com/molp11/SARS_HLA accessed on 8 August 2021.

## Supplementary Materials

The following supporting information can be downloaded at: https://www.mdpi.com/article/10.3390/life14091181/s1, Table S1: Patient HLA-type and Clincal Information; Table S2: HLA-A Peptide Binding Predictions; Table S3: HLA-B Peptide Binding Predictions; Table S4: HLA-C Peptide Binding Predictions
